# Piezoelectric and Magnetoelectric Effects of Flexible Magnetoelectric Heterostructure PVDF-TrFE/FeCoSiB

**DOI:** 10.3390/ijms232415992

**Published:** 2022-12-15

**Authors:** Dandan Wen, Xia Chen, Fuchao Huang, Jingbo Zhang, Pingan Yang, Renpu Li, Yi Lu, Yu Liu

**Affiliations:** 1 Doctoral Research Station of Chongqing Key Laboratory of Optoelectronic Information Sensing and Transmission Technology, Chongqing University of Post and Telecommunications, Chongqing 400065, China; 2 Chongqing Key Laboratory of Autonomous Navigation and Microsystem, Chongqing University of Post and Telecommunications, Chongqing 400065, China

**Keywords:** magnetoelectric composites, piezoelectric properties, PVDF-TrFE

## Abstract

Flexible polymer-based magnetoelectric (ME) materials have broad application prospects and are considered as a new research field. In this article, FeCoSiB thin films were deposited on poly(vinylidene fluoride-co-trifluoroethylene) (PVDF-TrFE) substrate by DC magnetron sputtering. The structure of PVDF-TrFE/FeCoSiB heterostructure thin films was similar to 2-2. Under a bias magnetic field of 70 Oe, the composites have a dramatically increased ME voltage coefficient as high as 111 V/cm⋅Oe at a frequency of about 85 kHz. The piezoelectric coefficient of PVDF-TrFE thin films is 34.87 pC/N. The surface morphology of PVDF-TrFE thin films were studied by FESEM, and the results of XRD and FTIR showed that the β-phase of PVDF-TrFE thin films was dominant. Meanwhile, the effects of different heating conditions on the crystallization and piezoelectric properties of PVDF-TrFE films were also studied. The flexible ME heterojunction composite has a significant ME voltage coefficient and excellent piezoelectric properties at room temperature, which allows it to be a candidate material for developing flexible magnetoelectric devices.

## 1. Introduction

Technology development in wearable electronic devices, sensors, and other fields has promoted the research progress of ME materials [1,2,3]. The composite system has better ME response than a single-phase ME system at room temperature [4,5,6], so most of the research work is focused on the composite ME system. Under the action of a magnetic field, the magnetostrictive phase produces strain, and the corresponding stress is transferred to the piezoelectric phase. Due to the inverse piezoelectric effect, voltage is generated on the piezoelectric material. This is called the direct ME effect (DME) [2,3,4,5].

Magnetoelectric composites can be divided into two categories according to the type of piezoelectric material: ceramic-based and polymer-based. A piezoelectric ceramic matrix is generally inorganic ferroelectric material with a relatively high ME voltage coefficient [7,8]. For example, Gao et al. investigated an asymmetrical bilayer Metglas/Pb(Zr,Ti)O_3_ with multi-push pull configuration, and obtained an ME voltage coefficient of 250 V/cm⋅Oe [9]. Ceramic-based composites not only have the disadvantages of a complex preparation process, but they are fragile and difficult to mold. At the same time, ceramic-based composites are also limited by the reaction in the interface region, resulting in high dielectric loss and low resistance, and it is difficult to process into mechanically compliant structures [10,11,12,13]. As a contrast, piezoelectric polymers have simple, scalable, and low-temperature manufacturing methods, such as hot pressing, solution casting, and annealing [14,15,16], which overcome the shortages of the ceramic-based composites. Poly(vinylidene fluoride) (PVDF) and its copolymers poly(vinylidene fluoride-cotrifluoroethylene) (PVDF-TrFE) and poly(vinylidene fluoride-co-hexafluoropropylene) (PVDF-HFP) are typical piezoelectric polymers [17]. PVDF is a semicrystalline polymer, because of its multifunctional properties, such as piezoelectric, pyroelectric, and ferroelectric properties. PVDF and its copolymers are widely used in energy storage, energy harvesting, capacitors, and other fields of modern electronic equipment. PVDF is characterized by four main polymorph phases, namely α, β, γ, and δ [18,19,20]. The β-phase of the PVDF polymer presents the highest dipole moments, and exhibits the highest piezoelectric and ferroelectric properties [20,21,22]. Compared with PVDF films, PVDF-TrFE can obtain a better piezoelectric response. Therefore, PVDF-TrFE can be considered as one of the most useful ferroelectric polymers with good mechanical flexibility [23,24].

To endow ME coupling in PVDF-TrFE-based polymers, magnetostrictive materials must be introduced. Researchers have studied the ME effect in various polymer-based composite systems. Polymer-based ME composites are often divided into 2-2 laminate type (consisting of horizontal ferroelectric and magnetic layers) and filling type (0-3 particle-loaded type and 1-3 fiber-loaded type), where each number represents the dimension of each phase [17,25,26]. Liu et al. developed polymer-based ME composites films, composed of magnetically oriented polycrystalline iron fiber (${\mathrm{Fe}}_{f}$) and PVDF-TrFE; a peak ME value of 44.5 mV/cm⋅Oe was observed for the film [27]. Silva et al. reported the effect of the bonding layer type and piezoelectric layer thickness on the ME response of layered PVDF/epoxy/Vitrovac composites, with the highest ME response of 53 V/cm⋅Oe [28]. The vast majority of the literature shows that the ME voltage coefficient of the laminated composite material is higher than that of the filled composite material. Considering the good mechanical flexibility of the film, in this article an amorphous alloy FeCoSiB was prepared on the PVDF-TrFE piezoelectric layer by magnetron sputtering to form a composite structure similar to 2-2.

In many amorphous alloy systems, FeCoSiB films have excellent soft magnetic properties, high magnetic permeability, and good magnetostriction coefficient [29,30,31]; therefore, in this work, the FeCoSiB sputtered layer has been selected as the magnetic layer in the ME heterostructure. At the same time, the ME voltage coefficient of the PVDF-TrFE/FeCoSiB heterostructure can reach 111 V/cm⋅Oe at a frequency of 85 kHz, which is much higher than ferrite composite and nanoparticle-filled composite. Although the composite based on Metglas reported a higher ME coefficient, magnetron sputtering-based ME composites can balance flexibility and brittleness, which is also a significant advantage. From the perspective of application, it is more suitable for flexible storage and energy collection applications.

## 2. Results and Discussion

**Figure 1:**
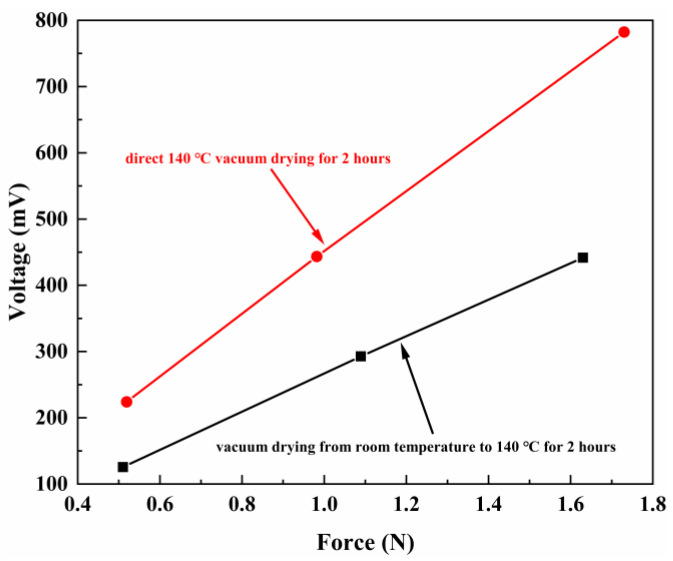
Voltage of the PVDF-TrFE films with different heating conditions versus the applied pressure.

In polymer-based ME materials, the electrical polarization of the piezoelectric phase will be changed arising from a deformation of the magnetostrictive phase, which leads to the variation of the surface potential. Thus, it is necessary to discuss the piezoelectric property of PVDF-TrFE. The piezoelectric coefficient (d_33_) is a measure of the magnitude of the internal electric field under unit mechanical stress (direct piezoelectric effect). At room temperature, PVDF-TrFE films were polarized at 6 kV and lasted for 400 s, and the d_33_ of the PVDF-TrFE films with different temperature increase conditions were recorded in Table 1. The results showed that d_33_ increased from 21.27 to 34.98 pC/N after vacuum drying at 140 °C for 2 h under different heating conditions. In addition, all the PVDF-TrFE films show a good linear relationship between the response voltage and the applied pressure, which is plotted in Figure 1. Meanwhile, we note that the slope of d_33_ of direct 140 °C vacuum drying for 2 h (named heating condition 2) is greater than that of vacuum drying from room temperature to 140 °C for 2 h (named heating condition 1). How heating conditions affect d_33_ will be discussed in the next section with additional experimental results.

Furthermore, it can be found from Table 1 that the d_33_ of PVDF-TrFE films measured before polarization were 0.24 pC/N and 0.34 pC/N respectively. After polarization, the d_33_ constants dramatically increased to 21.27 pC/N and 34.87 pC/N. It was reported that polarization of PVDF-TrFE films under an electric field can produce preferential orientation of the ferroelectric domains, thus leading to electroactive properties [32]. According to this theory, it can be inferred that in the experiment, polarization makes electronegative ions uniformly deposit on the surface of the sample, and generates a large electric field between the electrode and the bottom of the sample, forming an effective polarization, which explains the significant difference of d_33_ before and after polarization. The excellent piezoelectric properties can bring higher sensitivity in applications such as pressure sensors. The high piezoelectric coefficient obtained in this experiment is worthy of attention.

**Figure 2:**
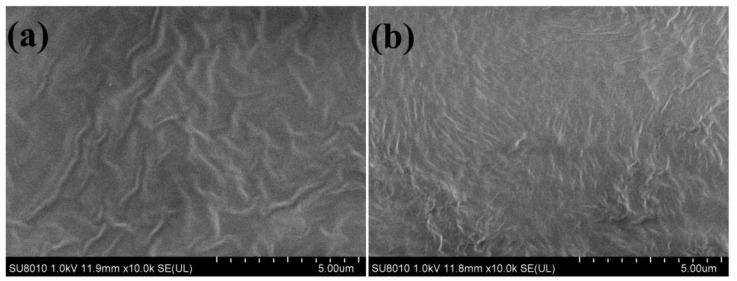
The FESEM images of PVDF-TrFE films at 10K magnification were used to analyze the surface topography of (**a**) heating condition 1, (**b**) heating condition 2.

The surface topography of polarized PVDF-TrFE films were analyzed using the FESEM analysis. The morphology of PVDF-TrFE films under different heating conditions is shown in Figure 2. From Figure 2, it can be seen that the surface of PVDF-TrFE film presents a wrinkled and stacked lamellar morphology under both conditions. Based on Li et al.’s research, we believe that this surface morphology is due to the presence of a large number of grains composed of rod-like crystallites in the film, which is a typical feature of the β-phase on PVDF-TrFE [16].

The significant development of the elongated microcrystalline structure was clearly observed from Figure 2. Compared with the sample at heating condition 1 (Figure 2a), the PVDF-TrFE film at heating condition 2 (Figure 2b) had a more compact microcrystalline arrangement and a more elongated crystalline region. At the same time, the formation of elongated crystallite structure enhanced the crystallinity properties of PVDF-TrFE thin films in Figure 2b. The reason for this phenomenon is that the curing temperature of the sample is different. The sample in Figure 2a is gradually heated from room temperature to 140 °C, which results in the curing temperature of the sample in Figure 2a as much lower than 140 °C, while the curing temperature in Figure 2b is always 140 °C. According to previous studies, the temperature between *T_c_* and *T_m_* will induce the formation of microcrystals [33]. The curing temperature (140 °C) of the sample in Figure 2b is higher than the Curie temperature (*T_c_* = 133 °C), but lower than the melting temperature (*T_m_* = 145 °C), so it is speculated that the curing temperature of 140 °C can provide strong crystallinity. This also explains the high crystallinity of the sample dried at 140 °C for 2 h. Generally speaking, the piezoelectricity depends on the crystallinity and the content of piezoelectric β phase, which can be used to explain the increase of d_33_ in the PVDF-TrFE membrane under different heating conditions after polarization.

**Figure 3:**
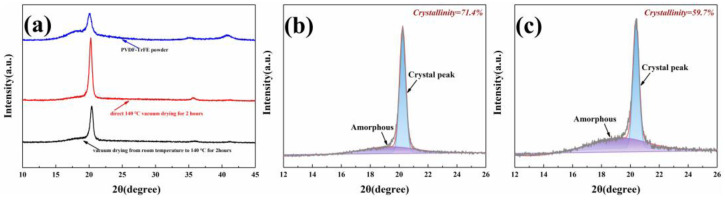
(**a**) X-ray diffraction curves of PVDF-TrFE powder and PVDF-TrFE films at different heating conditions; (**b**) deconvolution of the X-ray diagram for the PVDF-TrFE dried in heating condition 2 between 2θ values 12° and 26°; (**c**) deconvolution of the X-ray diagram for the PVDF-TrFE dried in heating condition 1 between 2θ values 12° and 26°.

The structural characteristics of the composite membrane were analyzed by XRD. The results are shown in Figure 3a, showing the XRD patterns of the PVDF-TrFE powder and PVDF-TrFE membrane under different heating conditions. The reflection characteristics of the PVDF-TrFE α-phase are concentrated at 2θ of 17.7°, 18.3°, 20.0°, 26.6°, 33.2°, 35.9°, and 38.8° [20,21]. Therefore, in Figure 3a, the peak of the PVDF-TrFE powder observed at 18.8° can be attributed to the presence of the α-phase, but the α-phase has almost no piezoelectric effect [34]. Whereas the peaks near 20°, 36.0°, and 41° indicate the existence of the β crystal in the PVDF-TrFE films after vacuum drying. The XRD results show that compared with PVDF-TrFE powder, under the other two heating conditions, the intensity of the β crystal increased and the intensity of the α crystal decreased, suggesting that the α-phase transitions to the β-phase. The XRD intensity of the sample dried in heating condition 2 was obviously enhanced. Compared with the sample under heating condition 1, the peak position of heating condition 2 shifts slightly to the left, which is related to the slight increase of interchain spacing caused by Gaussian defects induced in the ferroelectric phase during the paraelectric–ferroelectric transition after annealing and cooling [16]. The XRD pattern can also be used to calculate the total crystallinity percentage ($X_{c}$) of the PVDF-TrFE membranes. $X_{c}$ can be calculated by the following formula:

(1) $$X_{c}=\frac{A_{c}}{A_{c}+A_{m}}$$

where, $A_{c}$ and $A_{m}$ are the area under the crystalline and amorphous regions obtained from the integral part of the corresponding diffraction peaks, respectively [35]. Figure 3b,c shows the deconvolution of XRD patterns of PVDF-TrFE membranes with Gaussian curves related to the β-phase and amorphous phase. The crystallinity percentage of PVDF-TrFE membranes dried in heating condition 2 was calculated to be 71.4%, and the crystallinity percentage of PVDF-TrFE membranes dried in heating condition 1 was 52.06%. This is consistent with the results of FESEM images.

**Figure 4:**
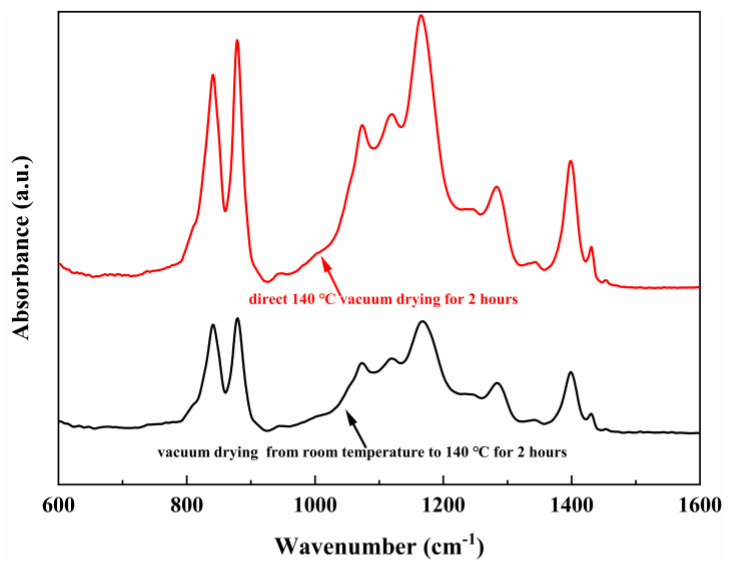
FTIR result of the PVDF-TrFE films with different heating conditions.

FTIR spectroscopy can be used to determine the polymer phases. Figure 4 shows the FTIR spectra of the polarized PVDF-TrFE film under different heating conditions.

According to the literature, the absorption bands at 840, 1280, and 1400 cm^−1^ are assigned to the electroactive β-phase [36]. The transmittance peak at 1400 cm^−1^ can be attributed to the CH_2_ wagging vibration, while the absorption bands at 840 cm^−1^ can be assigned to the CH_2_ rocking, symmetric stretching vibration of CF_2_, and the band at 1280 cm^−1^ for asymmetric stretching of CF_2_ [37]. Since the γ-phase also has weak piezoelectric properties, FTIR cannot clearly distinguish between the β-phase and the γ-phase, but there is no obvious band specific to the γ-phase in Figure 4, such as 1117 and 1234 cm^−1^. Therefore, in this spectrum, the three prominent transmittance bands observed at 840, 1285, and 1400 cm^−1^ corresponded to the polar β-phase, which indicates that the β-phase of all PVDF-TrFE films are dominant.

The peak value of PVDF-TrFE film dried in heating condition 2 is stronger, which is consistent with the results of XRD and FESEM images. It can be stated the film dried in heating condition 2 has enhanced the crystallinity and electroactive β-phase fraction. For PVDF and its copolymers, the piezoelectric properties depend on the polar electroactive phase. Therefore, it can be inferred that the PVDF-TrFE film dried in heating condition 2 has better piezoelectric properties, which is consistent with the above d_33_ results.

**Figure 5:**
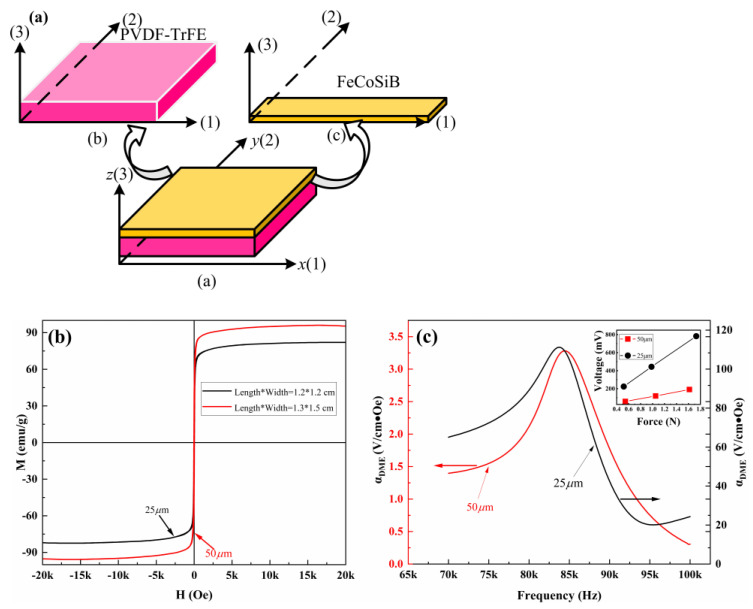
(**a**) Schematics diagram of PVDF−TrFE/FeCoSiB heterostructure similar to 2−2; (**b**) hysteresis loops of PVDF−TrFE/FeCoSiB composite films with 50 μm and 25 μm; (**c**) frequency dependence of the $\alpha_{DME}$ of PVDF−TrFE/FeCoSiB composite films with 50 μm and 25 μm in the frequency range from 70 to 100 kHz.

In this paper, FeCoSiB was sputtered onto the PVDF-TrFE membrane by magnetron sputtering to realize fully flexible double-layer ME composites; the schematic diagram is shown in Figure 5a.

To assess magnetism, the magnetization loops of PVDF-TrFE/FeCoSiB films were investigated in Figure 5b. The results of magnetization divided by the mass of the FeCoSiB films provide the specific magnetization with a unit of emu/g [38,39]. The formula m = ρ⋅v is used to infer the mass of the magnetic layer. Where, ρ is the mass density of 7.9 g/cm^3^ [38,39,40], v is the volume of the magnetic layer (length * width * thickness), and the thickness is inferred to be about 60 nm according to the sputtering rate. From the Figure 5b, it can be seen that all the composite films have a certain saturation magnetization and maintain the magnetism of FeCoSiB. The saturation magnetization of the samples are about 96 emu/g and 82 emu/g, respectively. It is lower than the saturation magnetization of pure FeCoSiB in the literature (~125 emu/g) [39,40,41]. This may be due to the fact that the PVDF-TrFE substrate is susceptible to stress and the PVDF-TrFE has a higher roughness than silicon substrate, resulting in more pinning centers. In addition, it may be due to the fact that the 50μm thick film surface is flatter, with a slightly higher saturation magnetization.

After confirming the stable piezoelectric properties with PVDF-TrFE and appropriate heating conditions, the ME response of composite samples with different thicknesses was studied. Figure 5c shows the frequency dependence of the $\alpha_{DME}$ of PVDF-TrFE/FeCoSiB composite films with different thicknesses in the frequency range from 70 to 100 kHz and relationship between applied stress and response voltage of PVDF-TrFE with different thicknesses. The $\alpha_{DME}$ initially increases linearly with *H_dc_* and begins to decrease after reaching the maximum. All composites have the maximum magnetoelectric coefficient near 83 kHz. The magnetoelectric coefficient peak of 111 V/cm⋅Oe was obtained for the sample with a thickness of 25 μm, which is much higher than the 44.5 mV/cm⋅Oe observed by Liu et al. in the PVDF-TrFE/Fe film [27]. This value is also about two times higher than the highest magnetoelectric coefficient observed in PVDF/epoxy/Vitrovac composites by Silva et al. [28]. At the same time, Figure 5c shows that the $\alpha_{DME}$ decreases significantly when the piezoelectric film thickness reaches 50 μm. This may be because the decrease of the ferroelectric behavior of the piezoelectric layer when the thickness of PVDF-TrFE increases to 50 μm, as shown in Figure 5c. Meanwhile, the ME voltage coefficient ($\alpha_{DME}$) is defined as the following formula:

(2) $$\alpha_{DME}=\frac{V_{p}}{t_{p}\cdot H_{ac}}$$

where $V_{p}$ is the induced voltage across the piezoelectric phase and $t_{p}$ is the thickness of piezoelectric material [42]. Therefore, it is speculated that the increase of piezoelectric layer thickness and the decrease of response voltage led to the decrease of magnetoelectric voltage coefficient.

The peak ME response of the sample with a thickness of 50 μm is 3 V/cm⋅Oe, which is still higher than that of ME particulate composites films composed of polymer and the magnetostrictive fillers. For example, the AC and DC ME coefficients of 69 mV/cm⋅Oe and 90 mV/cm⋅Oe has been reported for NiFe_2_O_4_-PVDF-TrFE [17].

## 3. Materials and Methods

N-methyl-pyrrolidone (NMP) as a solvent was provided by Macklin (Shanghai, China) and PVDF-TrFE was provided by Solvay (Brussels, Belgium). The (Fe_90_Co_10_)_78_Si_12_B_10_ alloy target was produced by DMxincai Company (Shijiazhuang, China).

PVDF-TrFE (80/20 mol%) powder was dissolved in NMP, the solution was stirred for 12h with a magnetic stirrer at room temperature, resulting in a homogenous transparent solution with 8 wt% PVDF-TrFE. We maintained a defined distance between the blade and the substrate to obtain a wet film thickness of ~600 or 1000 μm, and the solution was spread on the clean glass substrate to obtain a flexible film. At a controlled temperature of 140 °C, the solvent evaporates and the polymer crystallizes by annealing and drying in a vacuum oven for 2 h. Then, natural cooling to room temperature. Then, we made the PVDF-TrFE film polarize at room temperature with a self-made polarization device for 400 seconds. After polarization treatment, the FeCoSiB films were deposited on PVDF-TrFE substrates by DC magnetron sputtering. The back pressure was 0.5 Pa, the DC power was 110 W, the sputtering speed was 30 nm/min, the duration was two minutes, and the diameter of the FeCoSiB alloy target was 80 mm.

The d_33_ of the PVDF-TrFE films was measured using a d_33_ meter (self-made, ZILM Co. Ltd, Foshan, China). The morphology of the PVDF-TrFE films were evaluated via field emission scanning electron microscopy (FESEM, Hitachi SU8010, Tokyo, Japan) at an accelerating voltage of 1kV and 10K magnification. Phase formation and basic degree of crystallization of the PVDF-TrFE films were obtained by X-ray diffraction (XRD, Bruke D8 Advance, Karlsruhe, German) with Cu Ka radiation in the 2θ range of 10–40° and a scan rate of 5 °/min. Attenuated Total Reflectance (ATR) Fourier Transformed Infrared Spectroscopy (FTIR, Thermo Nicolet IS5, Waltham, MA, USA) is one of the most commonly used Fourier techniques. In the experiment, the polymer phase was evaluated by FTIR measurements, performed in the ATR mode at room temperature in the range of 600–1600 cm^−1^.

**Figure 6:**
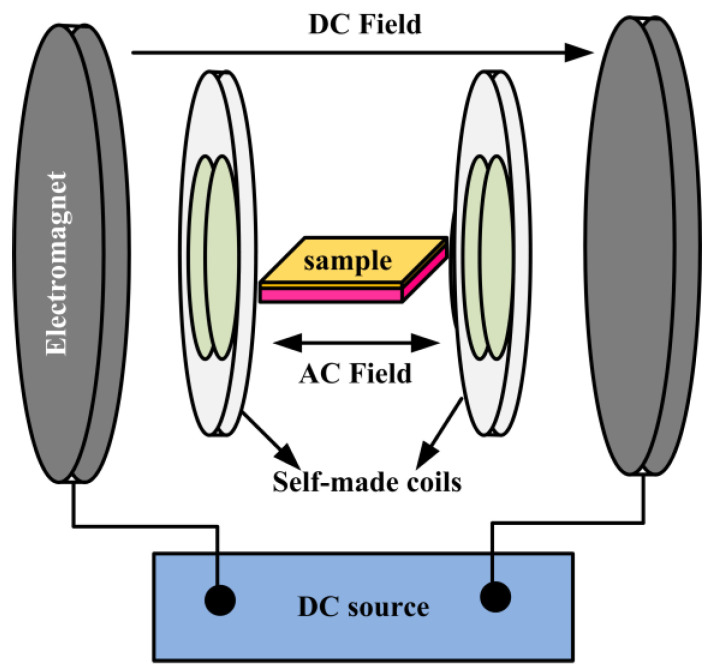
Schematic of the experimental setup for measuring magnetoelectric voltage coefficients.

The experimental setup for measuring the ME effect is shown in Figure 6. The AC magnetic field (*H_ac_ =* 0.5 Oe) generated by the self-made coil is superimposed on the DC bias magnetic field (*H_dc_* = 70 Oe) generated by the electromagnet. The composite film was placed in the center of the self-made coil, and the applied magnetic field was parallel to the film surface. The $\alpha_{DME}$ was studied using lock-in amplifier (Stanford research systems, Model SR830, Sunnyvale, CA, USA) and a computer was used for data collection. A vibrating sample magnetometer (VSM, LakeShore7404, San Diego, CA, USA) was employed to record the hysteresis loop under different magnetic fields; the magnetic field direction was parallel to the film surface.

## 4. Conclusions

In this paper, the ME composites based on PVDF-TrFE/FeCoSiB were prepared and studied. The peak ME voltage coefficient is about 111 V/cm⋅Oe at about 83 kHz. In addition, XRD and FTIR analysis of the polymer matrix indicated that the β-phase was dominant. It is found that the piezoelectric coefficient increases from 21.27 pC/N to 34.87 pC/N at different heating conditions. A feature of this study was to deposit FeCoSiB alloy on PVDF-TrFE substrate by magnetron sputtering to form a magnetoelectric heterostructure film with 2-2 structure. The structure retains the flexibility of the film, and it has been proved that it has a much higher ME voltage coefficient than the ME composites filled with magnetic fillers, which provides a new idea for the preparation and development of fully flexible magnetoelectric devices.

## Figures and Tables

**Table 1:** Piezoelectric coefficient (d_33_) of PVDF-TrFE films with different heating conditions before and after poling.

Materials	Before Polarization (pC/N)	After Polarization (pC/N)
PVDF-TrFE films with vacuum drying from room temperature to 140 °C for 2 h (named heating condition 1)	0.24	21.27
PVDF-TrFE films with direct 140 °C vacuum drying for 2 h (named heating condition 2)	0.34	34.87

## Data Availability

Not applicable.
